# Comparative Study of the Functional Outcome of Elbow Joint in Supracondylar Fractures of Children Treated With k Wire Fixation Using Lateral and Posterior Approaches

**DOI:** 10.7759/cureus.28232

**Published:** 2022-08-21

**Authors:** Adinarayana Kashyap, Abdul Ravoof, Harish Karigowda, Maheshwar Lakkireddy, Eppakayala Srikanth

**Affiliations:** 1 Department of Orthopaedics, All India Institute of Medical Sciences, Bibinagar, Hyderabad, IND; 2 Department of Orthopaedics, Adichunchanagiri Institute of Medical Sciences, B.G Nagara, IND

**Keywords:** displaced fracture, closed fracture, lateral approach, posterior approach, supracondylar humerus fracture

## Abstract

Introduction: Supracondylar fractures of the humerus are the most common elbow fracture reported in children. When closed reduction fails, open reduction and pinning will be used as a definitive procedure. In this study, we compare the functional outcome of lateral and posterior approaches of open reduction and assess the range of elbow movements and carrying angle by using Flynn's criteria.

Methods: This is a prospective study conducted on 30 children with supracondylar fracture of the humerus in Adichunchanagiri Hospital from a period of December 2018 to August 2020. A total of 30 children were selected for this study. Fifteen children underwent open reduction and pinning using the lateral approach (group 1) and 15 children underwent open reduction and pinning using the posterior approach (group 2) and all were followed up for six months. Functional outcome was assessed at the end of six months using Flynn's criteria.

Results: There were 20 boys and 10 girls in the study group. The mean age was 9.43 ± 1.69 years. The majority of children sustained injury due to falls on an outstretched hand (80%). One case of pin tract infection occurred in both the study groups. One patient had a superficial infection (6.7%) with the lateral approach, whereas two patients had a superficial infection with the posterior approach (13.33%). The lateral group fared better than the posterior group in comparison using Flynn criteria.

Conclusion: Lateral approach might be better than the posterior approach for unreduced supracondylar fractures necessitating open reduction and k wire fixation.

## Introduction

Supracondylar fractures are the most common elbow fracture reported in children comprising about 60% of fractures around the elbow in children and 13-15% of all pediatric fractures [1]. These fractures are common at 5-8 years of age in non-dominant hand and boys are more affected than girls [2-4]. Based on the displacement, these fractures are classified into three types. The fractured part is not displaced in type I, displaced with intact posterior cortex in type II, and completely displaced in type III, completely displaced with periosteal stripping in type IV [5]. Gartland type I fractures are usually managed non-operatively with an above elbow cast while Gartland type II and type III fractures are usually managed with closed reduction and percutaneous pinning. Fractures not amenable to closed reduction are treated surgically with open reduction and pinning. Open reduction can be done by anterior, posterior, medial, and lateral surgical approaches [6]. Some studies conclude that the lateral approach gives better functional outcomes while some other studies conclude that the posterior triceps sparing approach is safer and comparable to the lateral approach [7-9]. There is a constant debate over which approach is better in failed cases of closed reduction and pinning in supracondylar humerus fractures. The present study compares the functional results of supracondylar humerus fractures treated with k wire fixation using a lateral approach and posterior approach.

## Materials and methods

This is a prospective cross-sectional analytical study done in the Department of Orthopedics at Adichunchanagiri Hospital and Research Centre from December 2018 to August 2020 for comparing the functional outcome of the elbow joint in 30 admitted children diagnosed with a supracondylar fracture of the elbow treated with open reduction and internal fixation with k wires using a posterior approach in 15 children and lateral approach in 15 children.

Inclusion and exclusion criteria

Age less than 14 years and Gartland type III and type IV fractures. Exclusion criteria: Gartland type I and II fractures, open fractures, and supracondylar fractures with other associated injuries and neurovascular injuries.

Children that met inclusion criteria were divided into two groups on the basis of their Unique Health Identification (UHID) number. Odd numbers underwent a lateral approach and even-numbered patients were subjected to the posterior approach. The cases were operated by a single surgeon with more than 30 years of experience. All the children were assessed clinically and radiologically before the operative intervention and all the fractures were classified on the basis of Gartland Classification. Written informed consent was obtained from the parents/legal guardians for this study before the operative intervention. The pre-anesthetic evaluation was carried out and the patients were optimized before surgery. General anesthesia was used for all the patients. Written informed consent was taken and Injection Ceftriaxone 500 mg was given just before the procedure [7]. Functional outcome was assessed at the end of six months using Flynn's criteria [10].

Surgical approaches

Lateral Approach

Subjects were placed in the lateral decubitus position of the opposite side and the elbow is hung in the flexed position with soft support. After all the aseptic precautions, closed reduction was attempted first. In the event of failure of closed reduction, an incision measuring 5 cm was made over the lateral epicondyle. After dissection between the triceps muscle and the origin of the brachioradialis, the fracture site was exposed and open reduction was performed and the fracture was fixed with two parallel k wires in the lateral column under image intensifier guidance. The ends of the wires were left outside the skin for easy removal. Subcutaneous tissues were sutured with interrupted absorbable suture material and skin was closed with non-absorbable suture material. A long-arm posterior slab was applied.

Posterior Approach

Subjects are placed in the lateral decubitus position on the opposite side and the elbow is hung in the flexion position. Akin to the posterior midline approach, the skin and subcutaneous incision are made from 7 cm above to 2 cm below the olecranon. Subcutaneous tissues were dissected off the triceps muscle and fascia without splitting the muscle. The ulnar nerve is explored and safely preserved during the operation. The triceps muscle is dissected off both sides of the humerus (without splitting or cutting) and along the intermuscular septum exposing all the regions of the medial and lateral epicondyle, condyle, supracondylar ridge, and joint surface. Following the reduction of the fracture, fixation was done by cross pinning.

Postoperative period

The limb was immobilized in a long-arm slab immediately following surgery. Postoperatively limb elevation was given. Sutures/staples were removed on the 10th postoperative day. Active elbow mobilization was started at three weeks to prevent stiffness. Patients were followed up regularly at weekly intervals for four weeks postoperatively and were evaluated clinically for a range of motion at each visit. Radiological evaluation was done at three weeks, six weeks, 12 weeks, 18 weeks, and at six months following surgery for maintenance of reduction and union. K wires were removed within a 4-6-week period.

Sample size and statistical analysis

The sample size was calculated with a 95% confidence interval and a 10% margin of error was 30. SPSS version 22 (IBM Corp., Armonk, NY) and R environment ver. 3.2.2 were used for statistical analysis. Chi-square was used to assess the significance of study parameters.

Primary Outcome

The present study compares the functional results of supracondylar humerus fractures treated with k wire fixation done by lateral and posterior approaches using Flynn's criteria.

Secondary Outcome

The secondary outcome is the amount of blood loss and operative time period of the two approaches.

Chi-square was used to assess the significance of study parameters on a categorical scale between two groups and a p-value of <0.05 was taken to be statistically significant.

## Results

The mean age in this study was 9.43 ± 1.69 years. There were 20 (67%) boys and 10 girls (33%). The majority of children in this study sustained their injury due to a fall on an outstretched hand (80%). Right-sided supracondylar fractures (70%) were more than the left-sided supracondylar fractures (30%). Patients were surgically intervened within 24 hours and there was no significant difference in both groups. There was a significant difference in the blood loss in the posterior group (143.33 ± 19.88) as compared to the lateral group (92.53 ± 11.07). p-Value was ≤0.001 (Table 1).

**Table 1:** Comparison of associated blood loss in the study groups

Blood loss (mL)	Approach		Total
	Lateral	Posterior	
<100	14 (93.3%)	0 (0%)	14 (46.7%)
101-200	1 (6.7%)	15 (100%)	16 (53.3%)
Total	15 (100%)	15 (100%)	30 (100%)
Mean ± SD	92.53 ± 11.07	143.33 ± 19.88	117.93 ± 30.28

The lateral approach method was found to be quicker with a mean operative time of 54.06 ± 10.79 minutes as compared to the posterior approach (71.80 ± 9.91). p-Value was <0.01 (Table 2).

**Table 2:** Operative time comparison between the study groups

Operative time	Approach		Total
	Lateral	Posterior	
40-50	7 (46.7%)	0 (0%)	7 (23.3%)
51-60	3 (20%)	3 (20%)	6 (20%)
61-70	5 (33.3%)	5 (33.3%)	10 (33.3%)
71-80	0 (0%)	5 (33.3%)	5 (16.7%)
>80	0 (0%)	2 (13.3%)	2 (6.7%)
Total	15 (100%)	15 (100%)	30 (100%)
Mean ± SD	54.06 ± 10.79	71.80 ± 9.91	62.93 ± 13.60

There was one case of pin tract infection in the lateral approach group and there was one case of pin tract infection in the posterior approach group. Superficial infection was more in the posterior group (n = 2) when compared to the lateral approach group (n = 1) (Table 3).

**Table 3:** Wound/pin tract infection

Wound/pin infection	Approach		Total
	Lateral	Posterior	
NIL	13 (86.7%)	12 (80%)	25 (83.3%)
Yes	2 (13.3%)	3 (20%)	5 (16.7%)
Pin tract infection	1 (6.7%)	1 (6.7%)	2 (6.7%)
Superficial infection	1 (6.7%)	2 (13.3%)	3 (10%)
Total	15 (100%)	15 (100%)	30 (100%)

During our final assessments, all the patients have achieved fracture union. Results were assessed according to Flynn’s criteria (Table 4). 

**Table 4:** Functional outcome assessment using the Flynn's criteria

Flynn's criteria	Approach		Total
	Lateral	Posterior	
Excellent	13 (86.7%)	11 (73.3%)	24 (80%)
Good	2 (13.3%)	3 (20%)	5 (16.7%)
Fair	0 (0%)	1 (6.7%)	1 (3.3%)
Poor	0	0	0
Total	15 (100%)	15 (100%)	30 (100%)

**Figure 1:**
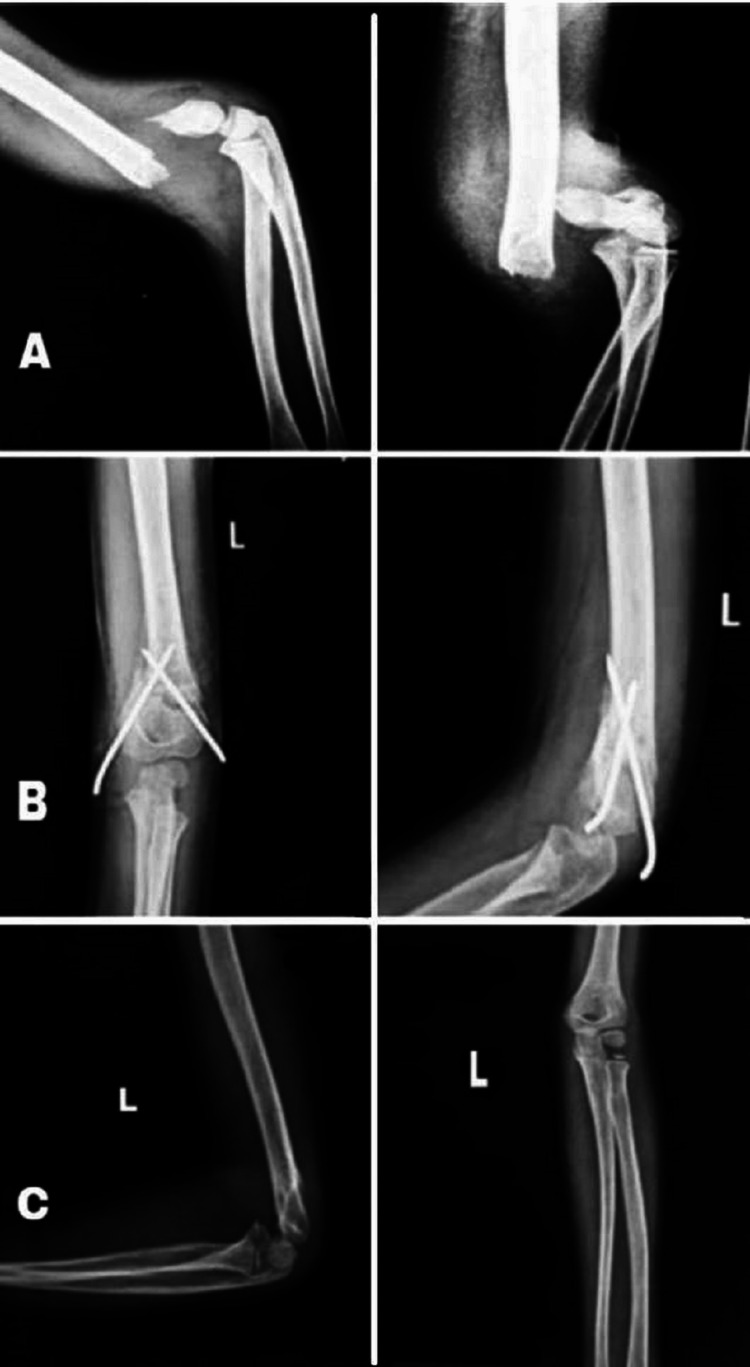
Radiological images of a nine-year-old female with type III supracondylar fracture. A. Preoperative supracondylar humerus fractures. B. At three-week postoperative period. C. At final follow-up of six months treated with the posterior approach

**Figure 2:**
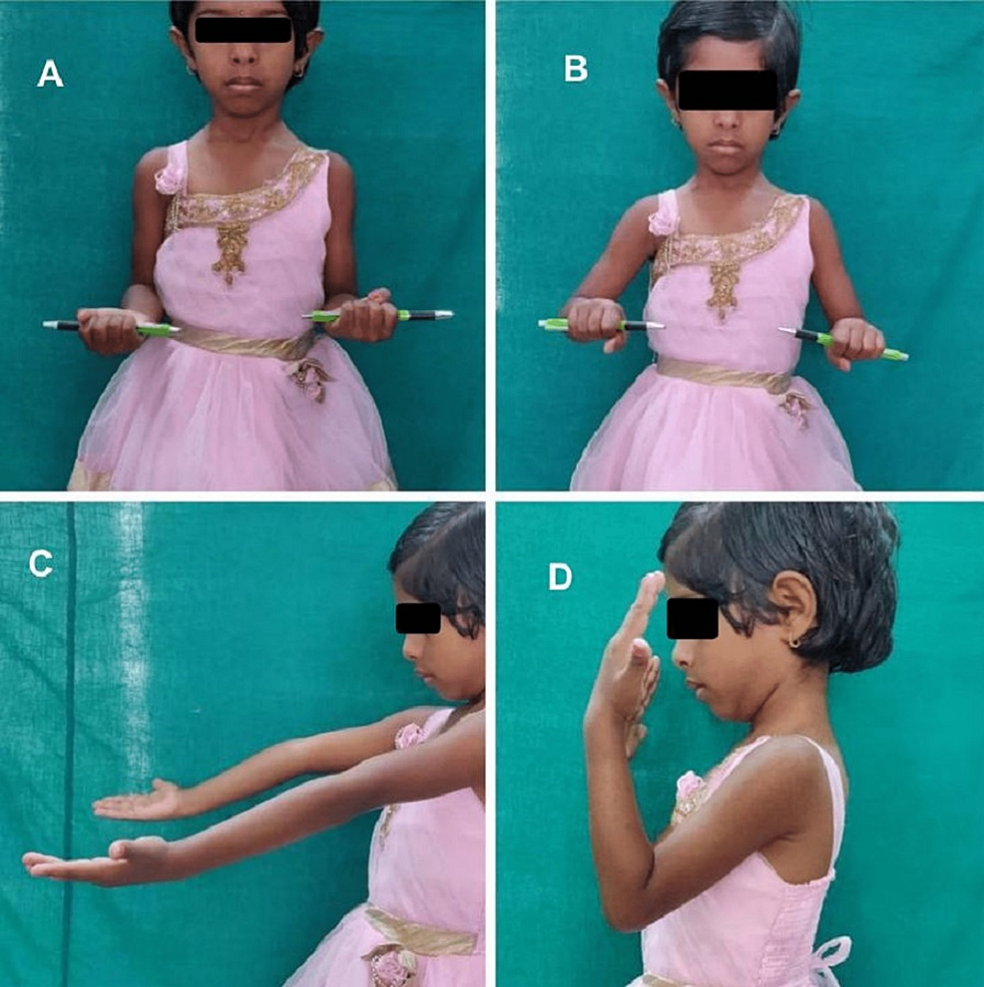
Range of elbow movements at six months of the postoperative period in a nine-year-old child operated with posterior approach. A. Supination. B. Pronation. C. Extension. D. Flexion

**Figure 3:**
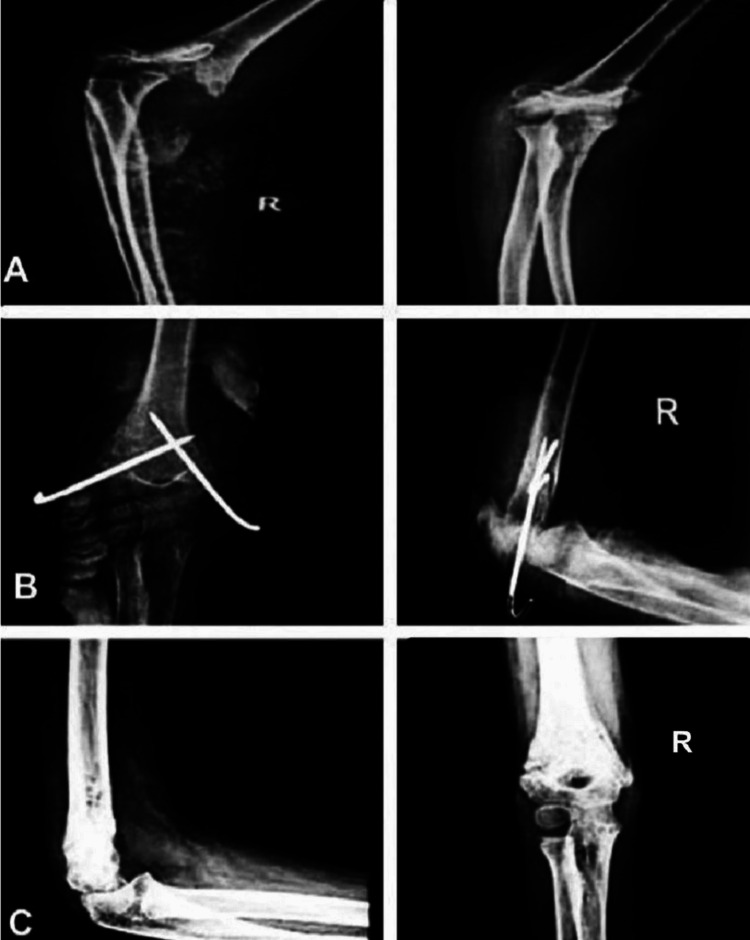
Radiological images of a eight-year-old with Gartland type III supracondylar humerus fracture. A. Preoperative supracondylar humerus fractures. B. At three-week postoperative period. C. At final follow-up of six months treated with lateral approach

**Figure 4:**
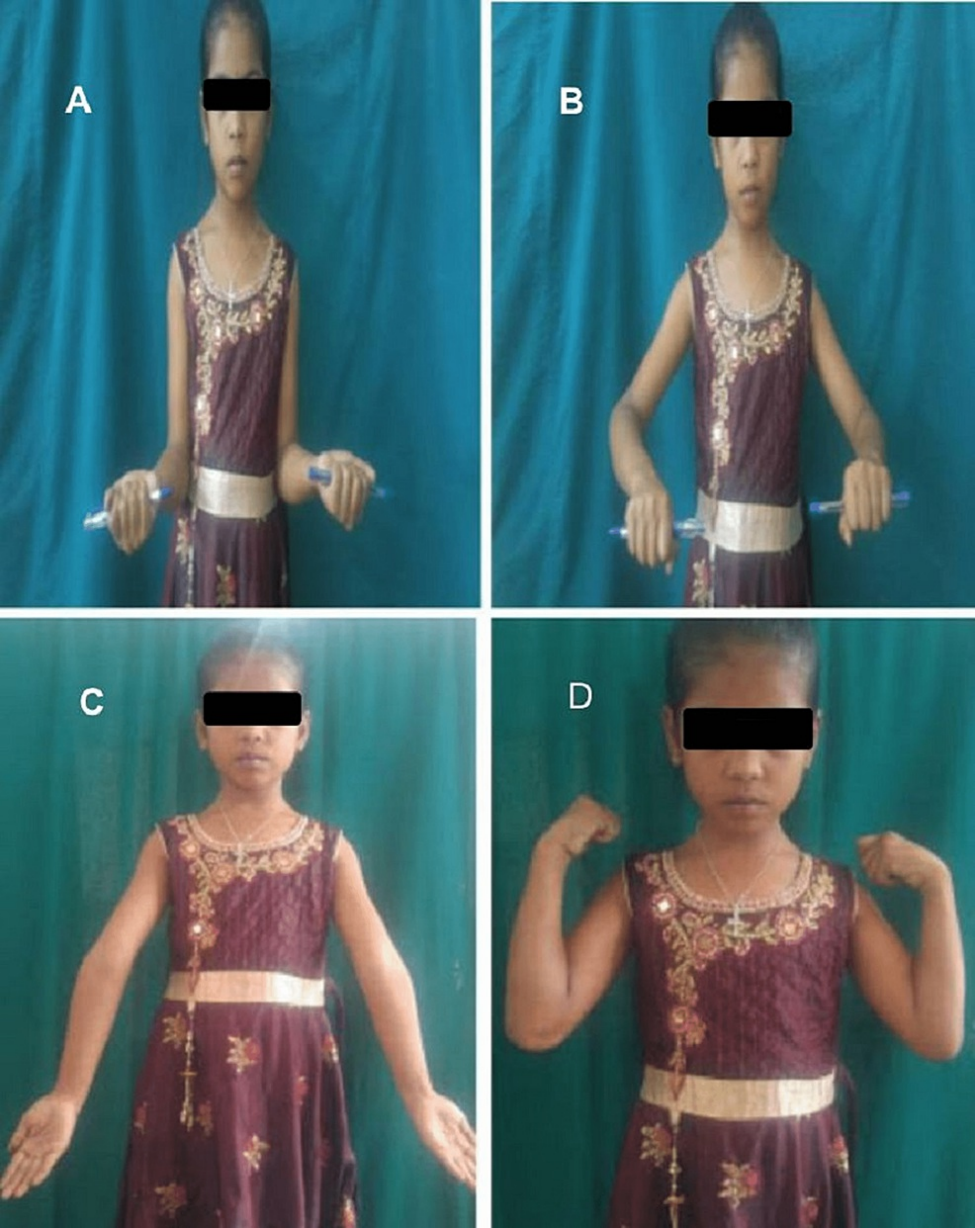
Range of elbow movements in an eight-year-old girl with type III supracondylar fracture of humerus at six months of postoperative period of the patient operated with lateral approach. A. Supination. B. Pronation. C. Extension. D. Flexion

Out of 15 children treated with a posterior approach (Figures 1, 2), 11 had excellent outcomes, three had good, and one patient had a fair outcome. Out of 15 patients treated with lateral approach (Figures 3, 4), 13 had excellent results and two had good outcomes.

## Discussion

Supracondylar fractures are one of the most common injuries in children. The aim of the treatment is to provide a functionally acceptable limb with a normal range of motion achieved at the earliest possibility [8]. Open reduction and internal fixation of Gartland type III and type IV supracondylar humeral fractures is an option utilized in failed closed reduction, open fractures, neurovascular injuries, increased edema, or hematoma.

In this study, children aged less than 14 years were evaluated. The mean age in this study was 9.43 ± 1.69 years, whereas it was 7.5 years in the study done by Waqar Alam et al. [8] and 7.8 years in a study by Ahmed and Mohmad [9]. This may be due to the small sample size of our study compared to the sample size of the above studies. There were 66.6% males in our study, which was comparable to the study done by Waqar Alam et al. on 153 patients, which had 63.4% males [8]. Twenty-one children sustained right-sided fractures, while there were nine children who sustained left-sided supracondylar humerus fractures. Abdul Wahab et al. in their study had 57% left-sided supracondylar fracture of the humerus [9]. A fall on an outstretched hand is the single most common cause of supracondylar humeral fractures. In this study, 80% of the patients sustained a fracture due to a fall on the outstretched hand, which is in consensus with the study done by Rahimi Shourin et al. [11].

Waqar Alam et al. in their study of 153 patients concluded that the lateral approach is the technique of choice although exposure with this approach is a bit difficult at times and it is easy to perform and less time-consuming. The final outcome was better when compared to the posterior approach [8]. In our study, there was a significant difference in the operative time period with the lateral approach being less time-consuming compared to the posterior approach (p < 0.01). This can be explained by the need for extensive dissection and exploration of the ulnar nerve, and preserving it increases the operation time significantly in the posterior approach.

Abubakir M Ahmed and Zaineb Abdul-Wahab Mohamad in their study of 36 children with supracondylar humerus fractures reported that the surgical treatment of extension supracondylar fracture of the humerus (type III) by lateral pinning approach has less related complications than the posterior approach [9]. In our study, two patients had superficial infections with the posterior approach and one patient had superficial infection with the lateral approach. There was one case of pin tract infection in both the lateral approach and posterior approach. There was no incidence of ulnar nerve injury in either of the study group. There was no incidence of malunion at the end of six months of follow-up.

In a study done by Komang et al., it is stated that the lateral approach is safer because less soft tissue is dissected, avoiding ulnar nerve damage. In cases requiring open reduction and internal fixation, the lateral approach is minimally invasive with minimal soft tissue dissection compared to the posterior approach. They, however, concluded by saying that the two approaches are comparable for treating supracondylar fractures in children when evaluated with Flynn’s criteria [12]. In the present study, 15 children out of 30 were treated with a posterior approach, 73% had excellent outcomes, 20% had a good outcome, and 7% patients had a fair outcome, and out of 15 patients treated with a lateral approach, 87% had excellent results and 13% had good outcomes. There were no poor results with both approaches in our study. The difference in the functional outcome between both approaches was not statistically different (p = 0.651).

Shahid Hussain et al. in their study concluded that the lateral approach for open reduction and internal fixation of widely displaced supracondylar fracture of the humerus is safe and straightforward, ensuring anatomical reduction and excellent function. It is safe and done through the internervous plane. This approach minimizes the chances of an inaccurate reduction [13]. The lateral approach is in a plane of the axis of the range of motion of the elbow and the posterior approach is perpendicular to it, whereas movement occurs in the plane perpendicular to the lateral approach. Therefore, fibrosis and adhesions might be more in the posterior approach leading to decreased range of motion as compared to the lateral approach. We had better results using Flynn’s criteria for the lateral approach (though statistically insignificant, p = 0.651 ) as compared to the posterior approach for displaced supracondylar fractures fixed with k wires after open reduction.

Ensafdaran et al. in their study have reported that the lateral approach, which involves a simple incision, causes less injury to the anatomical structures of the elbow, and the anterior and posterior parts of the fracture can be easily visualized, although it can be more difficult to manipulate the medial and lateral parts of the fracture at the same time and achieve good reduction [13,14]. They attributed this to damage to intact posterior structures which leads to decreased range of motion and so decreased functional results. They concluded that the lateral approach is better than the posterior approach with lesser complications and better long-term functional results [7]. Similarly, Waqar Alam et al. and Abubakir M Ahmed in their studies concluded that the lateral approach is better than the posterior approach [8,9]. But in our study, the difference in the functional outcome between both approaches was not statistically different. However, according to the systemic review performed by Mazzini et al., there is no consensus on the most acceptable approach along with the least complications in supracondylar fracture of humerus in children [15].

Limitations of the study: Long-term follow-up was not available as children were followed for a period of six months postoperatively. The study included a small number of patients.

## Conclusions

Supracondylar fractures in children require proper management. Surgery (open reduction and internal fixation with k wires) is required when closed reduction is not successful. Lateral approach while doing open reduction and fixation for management of Gartland type III supracondylar fracture in children is easy to perform and less time-consuming. It has lesser blood loss when compared to the posterior approach. The final functional outcome (Flynn’s criteria) is also better with this approach. 
